# Spectrum Occupancy Model Based on Empirical Data for FM Radio Broadcasting in Suburban Environments

**DOI:** 10.3390/s21124015

**Published:** 2021-06-10

**Authors:** Ajalawit Chantaveerod, Kampol Woradit, Charernkiat Pochaiya

**Affiliations:** 1 Center of Excellence in Sustainable Disaster Management, Department of Electrical Engineering, School of Engineering and Technology, Walailak University, Nakhon Si Thammarat 80161, Thailand; 2 OASYS Research Group, Department of Computer Engineering, Faculty of Engineering, Chiang Mai University, Chiang Mai 50200, Thailand; 3 Department of Electrical Engineering, School of Engineering and Technology, Walailak University, Nakhon Si Thammarat 80161, Thailand; pcharern@wu.ac.th

**Keywords:** spectrum measurement, spectrum sharing, public safety band, cognitive radio, dynamic spectrum access

## Abstract

It is well-known that the analog FM radio channels in suburban areas are underutilized. Before reallocating the unused channels for other applications, a regulator must analyze the spectrum occupancy. Many researchers proposed the spectrum occupancy models to find vacant spectrum. However, the existing models do not analyze each channel individually. This paper proposes an approach consisting (i) a spectrum measurement strategy, (ii) an appropriate decision threshold, and (iii) criteria for channel classification, to find the unused channels. The measurement strategy monitors each channel’s activity by capturing the power levels of the passband and the guardband separately. The decision threshold is selected depending on the monitored channel’s activity. The criteria classifies the channels based on the passband’s and guardband’s duty cycles. The results show that the proposed channel classification can identify 42 unused channels. If the power levels of wholebands (existing model) were analyzed instead of passband’s and guardband’s duty cycles, only 24 unoccupied channels were found. Furthermore, we propose the interference criteria, based on relative duty cycles across channels, to classify the abnormally used channels into interference sources and interference sinks, which have 16 and 15 channels, respectively. This information helps the dynamic spectrum sharing avoid or mitigate the interferences.

## 1. Introduction

The radio spectrum is part and parcel of wireless communication systems, such as television and radio broadcasting, satellite communication, mobile communication, a global positioning system (GPS), BlueTooth, and wireless local area network (WLAN). Federal government agencies in various countries organize these limited resources to meet users’ demands. Mainly, the spectra for radio broadcasting and mobile communication are more extensive than other activities due to their utilization. Many campaigns have been monitoring the broadcasting spectrum [1,2,3,4,5,6,7,8,9,10] to investigate the vacant range and reuse it for other applications. Their results point out that those spectra are underutilized, and other users should be allowed to access those spectra while they are unused. This spectrum sharing is referred to as the dynamic spectrum access technique. In addition, many researchers suggest that the spectrum utilization will increase when analog FM radio broadcasting is replaced with digital radio broadcasting. However, it is not guaranteed that digital radio broadcasting will be more popular with the audiences in the future. Some countries have delayed their plans to transit away from analog FM radio broadcasting to DAB, such as Sweden, Denmark, Thailand, and UK, because most people, who listen to radio are of the generations familiar with analog radio [11]. The government of each nation hesitates to migrate from analog FM radio to digital radio because a large budget is required for upgrading all transmitters and receivers. However, the spectrum for analog FM radio broadcasting in many countries is underutilized, and the spectrum utilization efficiency must be improved urgently. Thus, the spectrum of analog FM radio broadcasting is very attractive to a wide range of secondary users who need to access this spectrum for other activities, such as insertion of DAB, cognitive IoT, mobile communications, GPS, and Wide Area Network. For example, wireless wide area networks (Wireless WAN) with low power transmission for internet service are established in suburban and rural areas, where cellular networks have no coverage.

Many researchers have investigated advanced technologies to access unused spectrums for FM radio broadcasting. Reference [12] proposed the theoretical technique to access the FM radio spectrum for facilitating the communication between the secondary users. Later, the technique was implemented in Reference [11], where the prototype with Frequency Spread Filter Bank Multicarrier (FS-FBMC) transmitters was designed. Their experimental results pointed out that the FBMC technique can be developed in the future into an SU IoT Wide Area Network broadcast channel, which has many applications, such as energy smart grid balancing, rural communities, and emerging smart city technologies. To enhance the spectral efficiency in next-generation mobile communication, Reference [13] presents the single radio access architecture that supports both FS-FBMC and orthogonal frequency division multiplexing (OFDM). Their demonstration shows that the hardware of OFDM can be easily implemented and can also be used for FS-FBMC to reuse the OFDM resources. Reference [14] proposed the theoretical algorithm to determine the unused FM radio spectrum across the continental United States. It indicated the locations, which are promising to achieve their full potential usages by sharing the spectrum with the Cognitive Internet of Things (C-IoT) applications. Reference [15] pragmatically determined the unused spectrum across South Korea with channel allocation methods to allocate digital audio broadcasting (DAB) service channels into the frequency band of analog FM radio broadcasting. Reference [16] presents the FM-based positioning system and demonstrates them in two different areas, including the campus of National Taiwan University (NTU) and Wenshan rural area. The FM-based positioning system performs better than the GSM-based system and is beneficial for a rural area where the GSM base stations cannot fully cover the area. However, accessing the broadcasting spectrum confronts severe limitations due to the interferences resulting from several analog broadcasting stations [17], such as overrated carrier power, out-of-band emission, harmonic emission, frequency error, and intermodulation product. Consequently, implementing the dynamic spectrum access in broadcasting frequency bands relies on the technique to classify the frequency band’s state, whether it is really occupied by a broadcasting station or only by interference. The classification can be based on either deep learning [18] or deterministic criteria.

The researchers have developed techniques for sensing the occupying state of a frequency band. The measurement campaigns based on those sensing techniques pointed out that many frequency bands were underutilized and encouraged the regulators to modernize spectrum sharing by replacing the static spectrum access with the dynamic spectrum access. The spectrum utilization efficiency has been heavily studied since 1977 [19] to rearrange spectrum allocation. Matheson’s technique in Reference [20] is one of the popular techniques used for investigating the spectrum utilization, and its advantages are applied for modeling spectrum occupancy and evaluating the interference. Reference [21] classified the spectrum occupancy monitoring into five approaches, namely spectrum measurement methods [20], spectrum occupancy modeling [22,23], spectrum sensing [24], interference map creation [25,26,27], and spatial spectrum measurements [28]. The spectrum measurement methods are the basis for all other approaches and will be the focus on this work. Most spectrum measurement methods are based on the “electro-space” concept, which considers the signals of interest under the set parameters, namely frequency, location, direction, polarization, and time. Their important application is to devise the measurement strategies for different purposes: (i) investigating the spectrum utilization to perform regulation [20], (ii) characterizing spectrum activities [9,29], and (iii) establishing dynamic spectrum sharing with cognitive radio (CR) system.

We observed that there are two knowledge gaps in the literature. First, although many methods to monitor radio spectrum occupancy have been proposed, Reference [21] implies that their goal is little attentive to public broadcasting in suburban environments. Second, the preceding campaigns that survey the spectrum occupancy over FM radio band employ the general-purpose measurement techniques, not the focused-purpose technique. Since selecting different strategies leads to significantly different results, the focused-purpose technique should be emphasized [19,30]. Accordingly, we design the focused-purpose spectrum measurement strategies for FM radio broadcasting in suburban environments.

The essential information for the regulators to begin the FM radio spectrum reallocation is the visualized current utilization of the spectrum. A general-purpose spectrum occupancy model gives a less reliable utilization information because it ignores the different characteristics of the application in each frequency band. Specifically, the interference generated from older transmitters of the broadcasting stations is only found in the FM radio frequency band and is not taken into account by general models. Usually, the obtained utilization information fails to help the regulators distinguish the spectrum occupied by a primary user from that occupied by an interference. Therefore, the regulators need a new model that is particularly developed for the FM radio spectrum to obtain the more reliable utilization information. This paper aims to develop the spectrum occupancy model that is composed of the focus-purpose measurement strategies, decision threshold technique based on empirical data, occupancy classification, and interference classification. Thus, the paper’s central research objectives are:

- to modify the spectrum measurement strategies based on the concepts of References [20,21] and the recommendation of Reference [30] for monitoring the channel’s activity and distinguishing the power levels of the passband and the guardband separately,
- to devise the implementation of Reference [31]’s concept for analyzing the decision threshold with empirical data,
- to improve the accuracy in evaluating the spectrum occupancy of FM radio public bands by the proposed measurement strategies and threshold designation, as well as to gain insights into why the proposed strategies offer higher accuracy than the existing strategies,
- to gain insight into the pattern of spectrum occupancy of FM radio public bands in suburban areas, and
- to identify the channels, which cause interference, and the channels, which are affected by the interference.

To demonstrate the proposed spectrum occupancy model, we select a suburban area where the conventional radio broadcasting system is still used but not 24 h a day. Moreover, the transmitting system in the suburban area frequently causes interference in some FM channels and other adjacent bands. Thus, the measurement campaign was conducted in Nakhon Si Thammarat province, Thailand. Most broadcasting stations are located densely, and a few of them are near the airport. When visualizing the spectrum occupation from the measured data with the conventional model, it is found that the number of the spectrums are being used more than licensed users. This is in contrast to our expectation that the spectrum occupation is underutilized, like in other countries [1,2,3,4,5,6,7,8,9,10]. Thus, we propose a new model to achieve the more reliable spectrum utilization visualization. The new model must be general enough so that it is applicable to other suburban areas, as well. Our model was designed in the way that the noise floor, which is usually dynamic, does not affect the performance to find the appropriate decision. Furthermore, it performs the channel occupancy classification to discover the channels that are interfered by other much higher power signals. Therefore, our model also assists the regulators in identifying the channels that generate interference, as well as the channels affected by that interference. Then, the regulation can be enforced to mitigate the interference. Moreover, the proposed strategies yield superior occupancy detection, where a greater number of unused channels can be detected. The unused channels, which are not founded by conventional strategies, are found by the proposed strategies, which can be applied in dynamic spectrum access by enhancing the spectrum sensing performance in cognitive radio systems [32].

The remainder of this article is structured as follows. Section 2 briefly describes the public FM broadcasting network in Thailand and the previous spectrum usage studies. Next, in Section 3, we present the measurement strategies and convert them to the high-performance spectrum analyzer’s machine code. We demonstrate the empirical model from the measured data to analyze the particular decision threshold in Section 4. We seek the concealed channels by assessing the passband and guardband of the occupied channels in Section 5. Then, we conclude the paper in Section 6.

## 2. Measurement Location

To improve the spectrum utilization of FM radio broadcasting, it is necessary to classify the occupied channel’s state, which can be either user occupied or interference occupied, by using the proposed spectrum occupancy model. To evaluate the model’s performance, we conducted the Thailand measurement campaigns, which operated analog FM radio broadcasting throughout the country. It serves as a mass communication channel where the government can send information to people more efficiently. It has been prevalent because of the low cost and availability since the 1940s. The passive urbanization and the severe inequality problem in most developing countries, such as India and Thailand, necessitate public broadcasting rather than mobile communication. Although the suburban and rural populations in Thailand are decreasing by 10 million people over the previous decade, there are still more than 30 million people. These people cannot access data on the internet via a smartphone, which has a high initial cost, as well as a monthly expense. Therefore, half of the Thai population living in suburban and rural areas have demanded public FM radio broadcasting.

**Figure 1:**
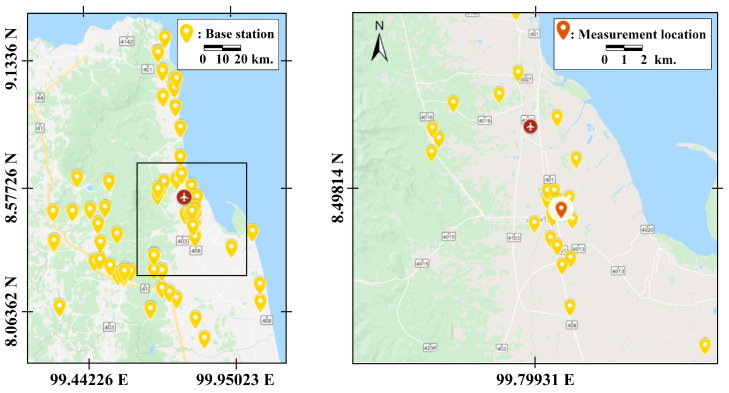
Locations of the public FM radio stations in the field measurements.

The public FM radio broadcasting network in Thailand covers over 76 provinces, and all of them are conventional analog systems. The frequency band used for FM radio is between 88–108 MHz and is split into 81 licensed channels. Each channel has a bandwidth of 250 kHz, including a passband of 150 kHz and two guardbands of 2 × 50 kHz. The whole country area is divided into 14 regions to reuse those 81 licensed channels. Each region has one radio monitoring control center. Nakhon Si Thammarat, which is an exception, has its own radio monitoring control center because its area is large (9.9 million square kilometers). Another reason is that the populations are dense at the center of the region since their living space locates between the Kao Lung mountain and the sea in the Gulf of Thailand. Figure 1 shows the locations of the public FM radio stations in the field measurements in the Nakhon Si Thammarat province of Thailand. The measurement location is in the downtown of Nakhon Si Thammarat province, in which the population is approximately 102,000, and its location is considered a suburban area. Since the coverage area is not large, the neighborhood often suffers interference from some stations transmitting too high power. Almost all the transmitting antennas are circular, and their height is limited to 60 m. The transmission power is limited to 500 watts and covers a radius of 20 km.

Previous studies in other countries pointed out that the FM spectrum was underutilized. However, when we used the conventional measurement strategy and the ITU threshold, our preliminary experimental results revealed that the FM spectrum in Thailand was not underutilized. This contradiction indicated that the threshold depends on the environment. It is necessary to analyze a new threshold to evaluate the spectrum occupancy. We hypothesize that some channels observed to be used might actually be occupied only by interference. Accordingly, it is of interest to be able to distinguish broadcasting activities from interference. In general, there are many noise sources, such as intermodulation, harmonic noise, and adjacent channels. These noise effects are more serious when the broadcasters keep a high signal to noise ratio by increasing the transmitter’s power. It affects the FM broadcasting network and disturbs other communication networks, such as the aeronautical system in the airband (108–137 MHz). Within a 20-kilometer radius of the airport, more than ten channels in the airband are frequently interfered. Moreover, within a 10-kilometer radius of the city center, intermodulation will occur with more than 70 channels in FM bands if all radio stations are active.

## 3. Measurement Setup

The existing spectrum measurement strategies in the literature are either for mobile communication [21] or for FM band but not specific to the environment [6]. In this section, we propose spectrum measurement strategies that can detect the occupied channels in public FM radio broadcasting. Although the spectrum occupancy measurements have various aspects, namely spectrum measurement methods, spectrum occupancy modeling, spectrum sensing, interference map creation, and spatial domain focus, the beginning step of these frameworks is the measurement setup. Most practical measurement setups, such as References [21,30], are based on the fundamental “Electro-space” concept of Reference [20]. They aid us to select the proper measurement setup carefully to obtain more in-depth knowledge. Since, the spectrum measurement is set up differently, it may lead to significantly different results. Here, we propose the different practical measurement setup based on the Electro-space concept by emphasizing more on the measurement strategy than the measurement system and converting it to the machine code which is used to control an appropriate automation system.

### 3.1. Measurement Strategy

To obtain the information for characterizing the pattern of spectrum occupancy and forming the criterion, which is used to distinguish the occupancy state between user occupied and interference occupied, we need the effective measurement strategies that are formulated with the focused-purpose technique, instead of the existing technique. This information containing the power level represented over time and frequency is essential for detecting the used spectrum and interference. The approach focusing on the time and frequency dimensions can provide sufficient information to estimate the spectrum occupancy due to the fixed spatial location of the FM radio broadcasting station.

When focusing on the frequency dimension strategies, Reference [30] points out that the estimation accuracy depends on the frequency span range and resolution frequency. Firstly, we monitor each channel and capture the power level when setting a frequency span equal to its transmitting signal bandwidth. Unfortunately, we cannot separate what is happening on the passband and the guardband. However, if the frequency span is narrower than the bandwidth (in this place, 250 kHz), we can classify the occupation whether it is in the channel’s passband or in the channel’s guardband because we can observe the activities within the passband and the guardband, separately. Then, we implement these guidelines in our experiment by setting the frequency span at 50 kHz to observe the power levels of 5 activities per channel in all 81 channels, totally 405 activities. Moreover, we improve the instrument’s sensitivity by adjusting the resolution bandwidth (RBW) in the strategy. When the RBW is narrow enough, the system can capture weak signals and decrease the noise floor. Accordingly, these strategies require a high-performance spectrum analyzer with a low noise figure and a high resolution.

For the time dimension strategies, References [21,30] defines the critical parameters for evaluating the spectrum occupancy, such as the measurement period and the sampling rate. The measurement period is a period of the representative estimate of the actual spectrum usage. Most previous campaigns of FM broadcasting modeled the duty cycle of the spectrum use with a period of 48 h. In their discussion, the preliminary experiment is recommended to check the validity of the selected period to avoid the underestimation and overestimation of spectrum utilization. Then, we made a preliminary inspection by measuring periodically in 48 h, and found that the measurement results were time-invariant. But, our results are not significantly different in the time domain. On the other hand, we could observe the different usage patterns when setting two 48-hour measurement periods on a weekday and a weekend. Then, we monitored the duty cycle of the spectrum use for four days within two months. Another aspect is the sampling rate, which is the rate that an instrument records Power Spectral Density (PSD) samples. We increased the sampling rate to collect the high resolution measured data, although it adds time processing. Therefore, these strategies necessitate a programmable instrument to operate the measurement routine automatically.

### 3.2. Measurement System

The designed measurement strategy requires a low noise figure and high sensitivity measurement system, as well as a large number of frequency points per span. To meet the requirements, we must employ a measurement system that works autonomously and quickly detects the spectrum occupied by the interference. Generally, the measurement system comprises the measurement devices and software for data processing. The measurement system can be developed based on either Software-Defined Radio (SDR) platforms or high-performance spectrum analyzers. For example, considering the FM band exploration, References [33,34] formulate the energy-sensing method with the Universal Software Radio Peripheral SDR platforms and programming languages based on Python and C++. These systems enhance the spectrum sensing decision process of CR, but only the high SNR signal is validated. As an example of the high-performance spectrum analyzer, References [35,36] captures the low SNR signal by enabling computer software to function on Virtual Instrument Software Architecture (VISA) and Standard Commands for Programmable Instruments (SCPI) softwares so that they realize the evaluation of the spectrum usage in a full frequency band.

#### 3.2.1. Sensing Setup

The implementation of the SDR platform is not a choice of the requirements of the proposed measurement strategies because their sensitivity is too low when capturing the power level among high interference. Then, we filter the high-performance spectrum analyzers which have the specifications in frequency and time dimensions, as well as the ability to process the spectrum automatically. For the strategies in the frequency dimension, the detailed specifications are (i) able to capture the weak signals among the noise in the frequency range of measurement study with marker tracking function, (ii) able to filter the signal in the frequency range of measurement study with the high-performance bandpass filter, and (iii) able to observe the spectrum of all the radio channels in an entire band. For the strategies in the time dimension, the detailed specifications are (i) able to simultaneously detect the presence of signals with very different power levels, (ii) able to routinely send collected data to the computer, and (iii) able to make a pre-processing for a large number of measured data. Accordingly, we choose a high-performance spectrum analyzer in the series of the Keysight NB9340B to construct the measurement device.

**Figure 2:**
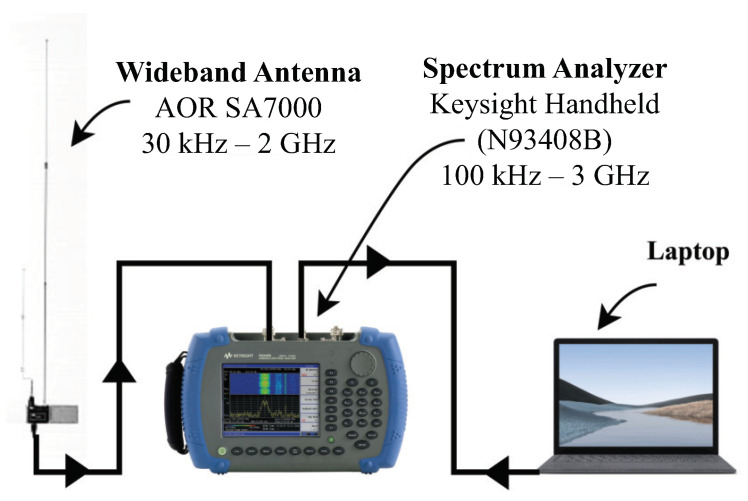
Testbed in the studied sensing scenario.

Figure 2 shows the testbed in the studied sensing scenario. The testbed consists of an antenna, a laptop computer, and a handheld spectrum analyzer. The antenna location is about 0.5–80 km from the base station of each FM radio station not necessarily with line-of-sight. The handheld spectrum analyzer is the Keysight NB9340B, which performs a measurement range from 100 kHz to 3.0 GHz and has a high SNR built-in preamplifier to capture the weak signal at each frequency bin by the average detection principle. It can be controlled with our command script and National Instruments (NI) Virtual Instrument Software Architecture (VISA) through the PC GPIB-USB port. The reception of the FM radio wave uses the AOR-SA7000 omnidirectional antenna, a passive arrangement providing two whip elements with a frequency range of 30 kHz to 2 GHz.

#### 3.2.2. Data Processing

With the performance of the Keysight NB9340B, it can estimate the spectrum over 87.375 to 107.875 MHz by using the proposed strategies and can process the large amount of power level data which represent the activities of all 81 channels. At a sample point of the measurement period, it directly monitors all the activities from 405 segments (81 channels × 5 segments) where each activity is investigated from the 50 kHz-width segment (as bandwidth of a channel equals 250 kHz). Within a channel, the passband and guardband have three and two acting activities, respectively. While monitoring the activity within a segment, it collects the 461 trace points of power levels, and the bandwidth between two consecutively measured frequency points, which is referred to as the frequency bin, equals 109 Hz. It spends approximately 1.003 s to capture 121,704 power level samples when setting the RBW at 3 kHz. When finishing one routine, the system determines the average value before recording it in the memory.

To obtain the measured data from the measurement system, we made the command script which was used to control the pre-processing on the high-performance spectrum analyzer and was used to characterize those data on the laptop before analyzing with the spectrum occupancy model in the next step. The first part in the command script built an interface between spectrum analyzer and laptop with serial number and established communication with the GPIB/USB port and VISA library. Next, the script had 410 loops which equaled the number of the segments in one measurement period. At the beginning of the loop, the script enabled the VISA Open Block to open a session for the device. Next, the command script adopted and sent the center frequency and frequency span to the instrument through VISA Agilent Write Block. The command script transferred the measurement parameters to the spectrum analyzer with following parameters: Attenuation 0 dB, RBW 3 kHz, VBW 3 kHz, Averaged Mode, and enabled Marker. They were encoded into the SCPI commands to set up the initial configuration of the instrument via VISA Write Block. After that, the device swept across the 461 frequency bins to capture the power levels. It took approximately 3.8 ms during one sweep. After capturing the data at the last frequency bin, the command script controlled the device repeatedly for 263 more times with the Time-Delay Block to achieve the next segment’s power level. After all 264 sweeps were complete, the system collected these data and saved them in an array (allocated memory) when the instrument received the SCPI command from VISA Read Block. Then, the script enabled the VISA Close Block. After finishing one loop, the system restarted the next loop immediately and so on until the last loop. Next, the command script exported the arrays of values from all measurement loops to a spreadsheet file. Finally, the communications of the spectrum analyzer and the instrument session were closed, respectively.

## 4. Decision Threshold Selection Criteria, Occupancy Classification Criteria, and Interference Criteria

This section determines a decision threshold, which is used to be a criterion for classifying the status of the spectrum occupancy. To improve the evaluation of spectrum utilization, numerous researchers pay attention to study the decision threshold, which is a function of the frequency band and the environment. Reference [30] categorizes the techniques to estimate the decision threshold in the literature into two main approaches, namely using prior knowledge of the noise properties and using advanced algorithms without any preliminary data. In addition, it points out that we should consider the tradeoff between required sensing time, complexity, and detection capabilities. The first technique creates the criterion for identifying the active status of a channel by a fundamental algorithm that does not require prior knowledge of noise. For example, in Reference [37], they select a threshold value for the given spectrum band with an iterative algorithm derived from One-Sided Hypothesis Tests (OSHT) in Reference [38]. Due to its high complexity, in practice, many measurement campaigns have preferred the techniques that require prior knowledge of the noise, described as follows. These techniques design the criterion based on the time series of various characteristics, such as signal power and noise power, together with the radio environment consideration. Even though this category’s techniques have a simple implementation, they require the crucial measurement device to detect the weak signal from noise. For example, in Reference [31], they measure the empirical data with a high-performance spectrum analyzer and study the usage pattern and the interference behavior in a band (from 850 to 870 MHz). This technique is promising because we attempted to apply this method for an FM band, and we found that the obtained threshold corresponds with that recommended by ITU. Accordingly, we decide to turn this method into a good account in analyzing the practical threshold.

### 4.1. Decision Thresholds in Previous Works

This subsection collects the decision thresholds used in earlier measurement campaigns to know the possible range of the thresholds for evaluating FM radio spectrum utilization. Almost all campaigns that survey and compare the spectrum usage over different frequency bands, such as FM radio band, radar band, maritime navigation band, TV broadcasting band, digital cellular services band, unlicensed Industrial, Scientific and Medical (ISM) band, wireless broadband access (WBA), and satellite band. They choose to use the single decision threshold either by following the ITU recommendation or by estimating noise characteristics in their radio environments. For example, the ITU recommendation in Reference [39] suggests that it should be 10 dB above the ambient noise. Reference [8] studied the spectrum usage pattern in Singapore in the frequency bands from 80 MHz to 5850 MHz in order to find the spectrum utilization over different services. They employed the constant threshold at 6 dB above the minimum detected signal power for evaluating the spectrum usage at the whole frequency range of the measurement study. Reference [10] presented the spectrum usage in a dense urban area near Washington, DC in the frequency band from 30 MHz to 3 GHz. They employed the threshold in the range between −100 and −105 dBm. These thresholds are applicable to the spectrum utilization comparison over various frequency bands, but they are not sufficiently reliable to model FM channels’ usage patterns. Therefore, we will benchmark the proposed threshold with the decision threshold recommended by ITU.

### 4.2. Proposed Implementation

The decision threshold analysis that is specific to the environment for mobile communications was proposed in Reference [31]. However, the given concept does not provide a concrete implementation in FM band. Therefore, we devise the implementation of that concept in FM band to determine the decision threshold in this subsection. The proposed technique helps obtain the decision threshold, which varies according to the channel activity and the interference, observed from empirical measurement data, to classify the channel occupancy more accurately. It consists of four steps. In the first step, we create a gradient map generated by the pixels presenting the measured data in order to indicate the range of possible values by glancing through this map. Second, the gradient map is transformed into a black/white map to explore each channel’s activities. When varying a threshold, we can easily classify the channels occupied full-time or part-time. Third, each channel’s occupation is modeled with the empirical data, and the results are plotted together with the duty cycle curve. By observing the curves’ characteristics, namely consistency and the sudden drop, we can identify the type of activities, namely strong, good, weak, and no activities, and group these channels according to their type. In addition, by observing the curve’s shape, we can classify the channels with high SNR from the channels with low SNR. Last, we analyze the set of possible decision thresholds by observing the interval on which all duty cycle curves, classified as high SNR, are constant, and identify the appropriate threshold for evaluating the spectrum occupancy in the next section.

#### 4.2.1. Creating Spectrum Density Map

The spectrum density map, a type of a heat map, is a technique of visualizing three-dimensional data where the intensity of color presents the measured signal power varying with frequency and time. First, we arrange the measured data in matrix form, as shown in Equation (1):

(1) $$\begin{array}{cc} P & =\left[{\tilde{P}}_{c,\tau }\right], \end{array}$$

where $c=1,\cdots ,N_{c}$ and $\tau =1,\cdots ,N_{\tau }$ are the index of the channel and the index of time instance, respectively, and ${\tilde{P}}_{c,\tau }$ is a row vector containing $N_{s}$ samples of power levels at channel *c* and time instance τ [expressed in decibels referenced to 1 mW (dBm)], which is expressed as

(2) $$\begin{array}{cc} {\tilde{P}}_{c,\tau } & =\left[P_{c,\tau }^{1}\;\;\ldots \;\;P_{c,\tau }^{s}\;\;\ldots \;\;P_{c,\tau }^{N_{s}}\right], \end{array}$$

where *s* is the index of the segment in each channel. In Section 4.3.1, the matrix P will be used to make the three-dimensional plot, referred to as a spectrum density map, which helps us distinguish the channels certainly occupied by a signal from the channels not certainly occupied by the signal, so that we can confidently indicate the channel occupancy.

Second, to search the threshold, it should be lower bounded by the noise floor to distinguish signal from noise. The noise floor can be estimated from the matrix P by

(3) $$\begin{array}{cc} \mathrm{Avg}(\mathrm{NF}) & =\frac{1}{N_{\tau }N_{c}N_{s}}\sum_{\tau =1}^{N_{\tau }}\sum_{c=1}^{N_{c}}\sum_{s=1}^{N_{s}}Min\left(P_{c,\tau }^{s}\right). \end{array}$$

In addition, the threshold should be upper bounded by the peak of the power level over all sampled data. The peak value can be estimated the matrix P by

(4) $$\begin{array}{cc} Max(P) & =Max\left[{\tilde{P}}_{c,\tau }\right]. \end{array}$$

The obtained lower bound and upper bound determine the range for searching the particular decision threshold, as will be shown in Section 4.3.3.

#### 4.2.2. Creating Channel Occupancy Map

The channel occupancy map visualizes three dimensional data where the black/white color presents the occupancy state, varying with the frequency and time. First, we arrange the state of occupancy in matrix form, as shown in Equation (5):

(5) $$\begin{array}{cc} Q & =\left[{\tilde{Q}}_{c,\tau }\right], \end{array}$$

where $c=1,\cdots ,N_{c}$ and $\tau =1,\cdots ,N_{\tau }$ are the index of the channel and the index of time instance, respectively, and ${\tilde{Q}}_{c,\tau }$ is a row vector containing $N_{s}$ occupancy state, which is represented as

(6) $$\begin{array}{cc} {\tilde{Q}}_{c,\tau } & =\left[Q_{c,\tau }^{1}\;\;\ldots \;\;Q_{c,\tau }^{s}\;\;\ldots \;\;Q_{c,\tau }^{N_{s}}\right], \end{array}$$

where *s* is the index of the segment in each channel, and $Q_{c,\tau }^{s}$ is an occupancy state of the segment *s* of channel *c* and time instance τ, which is defined as

(7) $$Q_{c,\tau }^{s}=\left\{\begin{array}{ccc} \; & 1\;if & P_{c,\tau }^{s}>\gamma \\ \; & 0\;if & P_{c,\tau }^{s}<\gamma , \end{array}\right.$$

where γ is a selected decision threshold. In Section 4.3.2, the matrix Q will be used to make the three-dimensional plot, referred to as a channel occupancy map, which helps us distinguish the channels occupied in full-time or part-time, so that we can make a hard decision on the channel occupancy state.

Second, to achieve the interval of the particular threshold according to the usage of the channels in the environment, we should exploit the prior knowledge about those occupied channels. For example, given that the occupied channel is a full-time case, the power signal is time-varying. Therefore, we calculate the total of the channel occupancy state ${\tilde{Q}}_{c,\tau }$, which can be expressed as

(8) $$\begin{array}{cc} A_{c} & =\sum_{\tau =1}^{N_{\tau }}A_{c,\tau }, \end{array}$$

where $A_{c,\tau }$ is defined as

(9) $$A_{c,\tau }=\left\{\begin{array}{cc} \; & 1\;if\;\;\;\sum_{s=2}^{N_{s}-1}Q_{c,\tau }^{s}>\left(N_{s}-2.5\right)\\ \; & 0\;if\;\;\;\sum_{s=2}^{N_{s}-1}Q_{c,\tau }^{s}<\left(N_{s}-2.5\right). \end{array}\right.$$

The interval of the particular threshold should be the interval of power signal levels that all channels have a constant occupation percentage. To be more efficient when classifying the type of activities, we select some channels instead of using all channels to get a mirror of diary activity, such as active and no activities, by using the obtained average value $A_{c}$ to select the channels occupied at full-time or part-time. The determination of the particular threshold interval will be demonstrated in Section 4.3.3.

#### 4.2.3. Modeling Channel Occupancy

**Figure 3:**
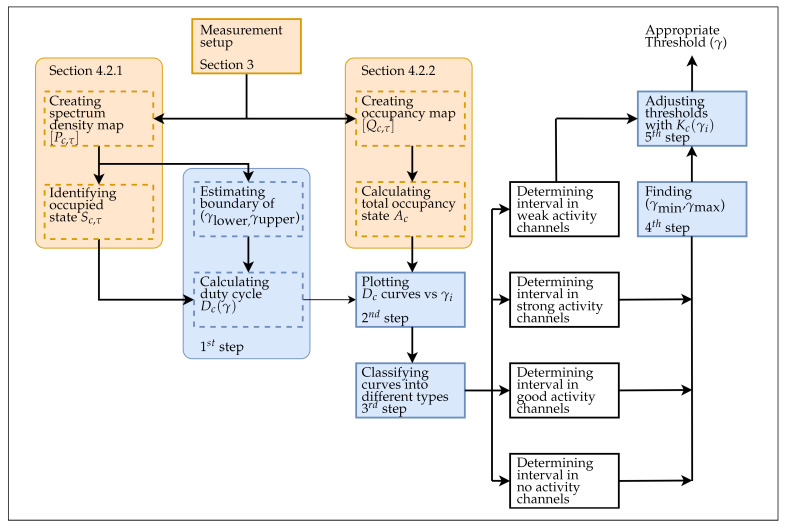
Process to determine appropriate decision threshold.

Generally, to quantitatively evaluate the channel occupancy, we use the duty cycle, which is the percentage of time that the channel is occupied, as a measure. To determine the duty cycle of a channel, a channel is considered being used at a given time if the channel’s power level is greater than a decision threshold. Accordingly, the appropriate decision threshold is critical to improving the accuracy of the channel occupancy model. We substitute the sum of durations *T*, the duration of time instance $\Delta T_{\tau }$ where τ is the index of time instance, and a occupancy state $S_{c,\tau }$ where *c* is the index of channel in Equations (10)–(12) to search for the optimal decision threshold, denoted by γ. Figure 3 presents a process to determine the appropriate decision threshold. The process contains the calculations of the variables in Section 4.2.1 and Section 4.2.2, as well as five main steps, which are described as follows. First, we formulate the duty cycle of each channel in Equation (10):

(10) $$\begin{array}{cc} D_{c} & =\frac{1}{T}\sum_{\tau =1}^{N_{\tau }}\left(\Delta T_{\tau }S_{c,\tau }\right), \end{array}$$

where $S_{c,\tau }$ is defined as

(11) $$S_{c,\tau }=\left\{\begin{array}{ccc} \; & 1\;if & P_{av,c,\tau }>\gamma \\ \; & 0\;if & P_{av,c,\tau }<\gamma , \end{array}\right.$$

where $P_{av,c,\tau }$ is the averaged power of channel *c* at time instance τ and is defined as

(12) $$\begin{array}{cc} P_{av,c,\tau }\; & =\frac{1}{N_{s}}\sum_{s=1}^{N_{s}}P_{c,\tau }^{s}. \end{array}$$

Second, we initialize the search for the decision threshold by randomly picking up a decision threshold γ to substitute in Equation (11), and make a plot of occupation percentage from Equation (10) as a function of power level. Note that the plot contains several curves, each of which represents each selected channel from Section 4.2.2. The interval of power levels that all curves have constant occupation percentages is the interval for choosing appropriate particular threshold. This interval can be determined as mentioned in Section 4.2.1.

**Figure 4:**
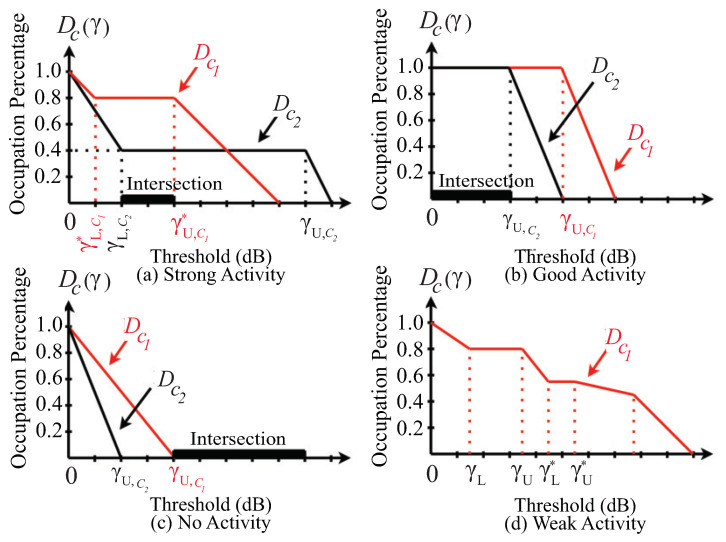
The shapes of the duty cycle curves are classified into four classes: (**a**) strong activity, (**b**) good activity, (**c**) no activity and (**d**) weak activity. Each curve has constant occupation percentage within the interval of the particular threshold where the lower and upper points are denoted as $\gamma_{L}$ and $\gamma_{U}$, respectively.

Third, before determining the interval of possible decision threshold, we select all channels with high SNR (insignificantly affected by interference) by observing two characteristics: (i) having a constant percentage over a long interval in the middle of the curve and (ii) transiting abruptly to zero near the end of the curve. Accordingly, we classify the channels, based on their duty cycle curves observation, into four classes: strong activity, good activity, no activity, and weak activity by the following criteria, as shown in Figure 4.

- A channel has strong activity if its curve has constant occupation percentage while the value of $D_{c}\left(\gamma \right)$ is between zero and one.
- A channel has a good activity if its curve has constant occupation percentage while the value of $D_{c}\left(\gamma \right)$ equals one.
- A channel has no activity if its curve has constant occupation percentage while the value of $D_{c}\left(\gamma \right)$ is zero.
- A channel has weak activity if its curve has constant occupation percentage over many intervals.

Fourth, we determine the interval on which all curves, classified as strong or good or no activity, are constant. In other words, when observing all the curves, classified as strong or good or no activity, the intersection of the constant intervals of all curves is the interval of particular threshold. Notice that we exclude the curves, classified as weak activity, from considering the interval of particular threshold since those curves are presenting the power level near the interference. Denote the obtained interval by [$\gamma_{min}$, $\gamma_{max}$].

Since the obtained interval has a constant duty cycle for the strong, good, and no activities, it is not guaranteed to be constant for the weak activity. Fifth, considering all channels classified as weak activity, we define $K_{c}\left(\gamma \right)$ as the number of weak channels whose duty cycles are not constant at a threshold of γ. The duty cycle curves are obtained by measurement, and hence the curves are discrete, and the differences are calculated to decide whether the curves are constant or not.

(13) $$\begin{array}{cc} K_{c}\left(\gamma_{i}\right) & =\sum_{c=1}^{N_{c}}\delta \left(\gamma \right), \end{array}$$

where $\delta (\gamma_{i})$ is defined as

(14) $$\delta \left(\gamma_{i}\right)=\left\{\begin{array}{cc} \; & 1\;if\;\;\;\Delta D_{c,\gamma_{i}}\neq 0\\ \; & 0\;if\;\;\;\Delta D_{c,\gamma_{i}}=0, \end{array}\right.$$

where $\Delta D_{c,\gamma_{i}}=D_{c}\left(\gamma_{i}\right)-D_{c}\left(\gamma_{i-1}\right)$. Then, we obtain the appropriate decision threshold as the $\gamma_{i}$ that gives the minimum $K_{c}\left(\gamma_{i}\right)$.

### 4.3. Results

This section analyzes the appropriate decision thresholds with the proposed technique in Section 4.2 and the empirical data obtained by using the spectrum measurement strategies in Section 3. In our measurements, we place the measurement device at the fixed location which is presented on the map in Figure 1. The measurement location is chosen in such a way that the device can detect the power spectrum of all channels. To observe the difference in spectrum usage patterns between weekdays and weekends, as well as that in different months, we placed the measurement device at the same location. We recorded the data on Sunday, the 22nd, and Monday, the 23rd, in September 2019, and on Sunday, the 27th, and Monday, the 28th, of October 2019. The data were collected once every five hours, giving us three sets of samples per day. Therefore, we obtained twelve sets of samples, which were sufficient for quantifying frequency usage.

#### 4.3.1. Spectrum Density Map

**Figure 5:**
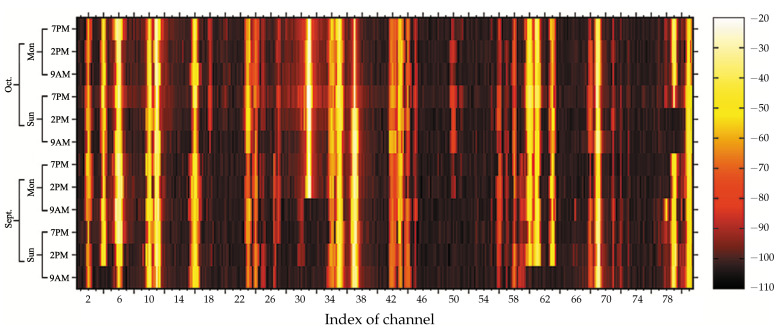
Spectral density map for the 81 channels of public FM radio broadcasting for twelve hours of the day.

We wrote the random signal power of all 81 channels, having a frequency of 87.375 MHz to 107.875 MHz, in matrix form, P with $N_{c}\times N_{s}$ rows and $N_{\tau }$ columns. In this particular case, the matrix P had 81×5=410 rows and 12 columns. Then, we made the spectrum density map, where each pixel corresponded to each element of the matrix P, as shown in Figure 5. The power levels were varying between −110 and −20 dBm. Two-thirds of all samples had the power levels that were greater than −98 dBm, and the maximum power level was of Channel 31. The noise floor Avg(NF) estimated by Equation (3) was −104.184 dBm, and the peak value of the power level Max(P) estimated by Equation (4) was −20 dBm. It was noticeable that a high power signal obviously occupied Channel 4, 6, 11, 31, 35, 37, 60, 61, and 69. On the other hand, a low power signal occupied Channel 21, 40, 49, 53, 65, 74, 76, and 77 and caused difficulty identifying the state of occupancy. Therefore, we exploited the obtained results, such as the average noise floor and the lower and upper bounds (−110 and −20 dBm, respectively) for setting the domain of the function $D_{c}\left(\gamma \right)$ while considering the search interval of decision thresholds.

#### 4.3.2. Channel Occupancy Map

**Figure 6:**
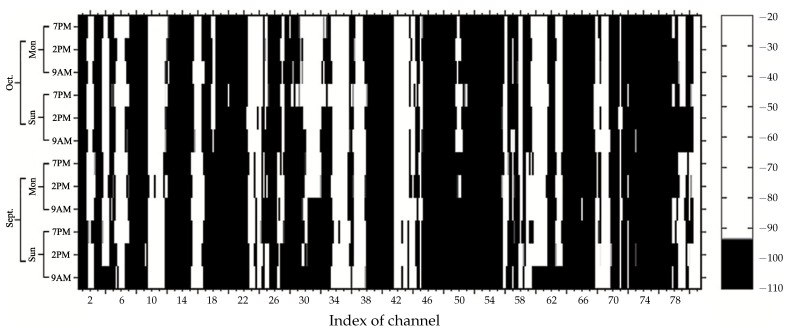
Channel occupancy with a threshold of 10 dB according to the ITU recommendation.

We determined the occupancy states of all segments in each channel by comparing the power levels with the standard threshold at 10 dB, recommended by ITU, and wrote them in matrix form, Q. The matrix Q had the same size as P. Each element in the matrix Q was 1 if the power signal at that segment was above −94 dBm, and it represented a white pixel of the occupancy map, as shown in Figure 6. Otherwise, the element was 0, and was seen as a black pixel. We classified the channel’s activity type by applying the decision threshold on the channel occupancy’s total. In this particular case, the channels *c* with an $A_{c}$, estimated by Equation (8), of 12 were classified as the full-time case, while the channels *c* with an $A_{c}$ between 1 and 11 were classified as the part-time case. In a full-time case, the black pixels represented on Channel 6, 11, 34, 35, 37, 43, 44, 68, 69, and 81 overall sampling times. In a part-time case, the alternating of black and white pixels represented on Channel 4, 9, 18, 31, 60, 61, 63, 66, and 79 overall sampling times. All channels in the full-time case and the part-time case were selected to plot the curves of occupation percentages and to exploit their power signals to gain prior knowledge about the occupied channels and to determine the search interval of the decision threshold through the curve of occupancy percentages in the next step.

#### 4.3.3. Curves of Duty Cycle

To achieve the appropriate decision threshold, we accessed the prior knowledge of noise properties and the environment through the duty cycle curves of all activities’ channels, including strong, good, weak, and no activities. We plotted the $D_{c}$ curve of each selected channel by substituting the power signals $P_{c,\tau }^{s}$ into Equation (10) and by substituting a decision threshold into Equation (11). These curves’ values monotonically decreased from 100 to 0% when substituting the γ from the lower bound to the upper bound. The duty cycle curves with all activities were plotted separately in four figures.

**Figure 7:**
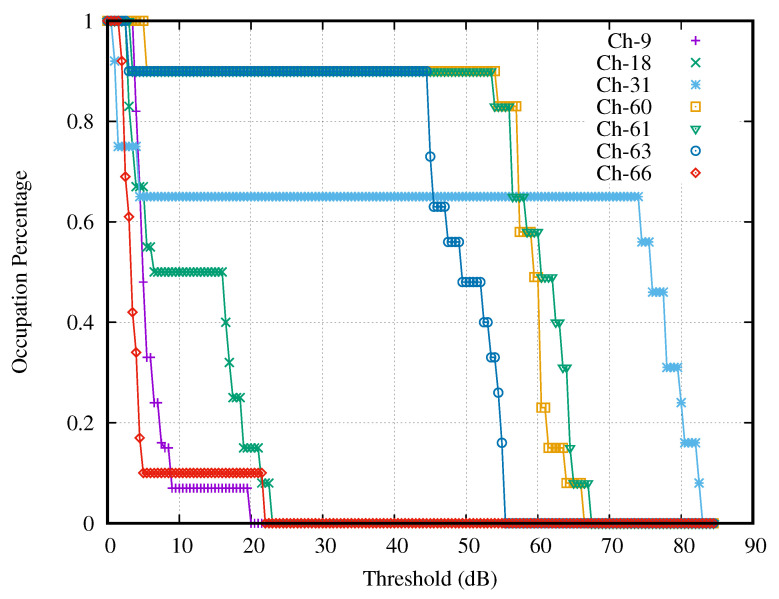
Channels with strong activities.

Figure 7 presented the curves of the selected channels having strong activity. These curves varied with the thresholds (dB), which were the difference between the testing thresholds (dBm) and the lower bound of the search interval (dBm). Notice that all selected channels gave the curves, which contained the steep slope because these channels had high SNR. It is noticeable that Channel 31 had the widest interval of a constant occupation percentage, whereas Channel 9 had the narrowest interval. At each curve, we observed the interval of threshold γ that made the occupation percentage curve constant. The intersection of the intervals of all curves served as the search interval of the optimal decision threshold. In this case, the search interval was 9–17 dB.

**Figure 8:**
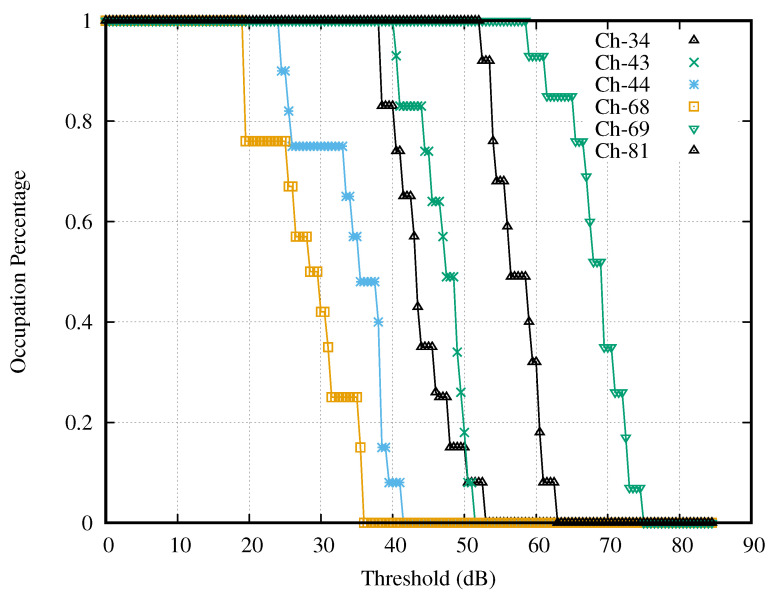
Channels with good activities.

Figure 8 presents the curves of the selected channels with good activities. It is noticeable that the curve of Channel 68 had the shortest interval of constant occupation percentage, i.e., 0–19 dB, which was longer than that of the strong activity case. Therefore, the search interval of the decision thresholds in the case of channels with good activity was the superset of that of the channels with strong activity, and the search interval of the decision thresholds in the case of channels with strong activity also served as the search interval in the case of channels with good activity.

**Figure 9:**
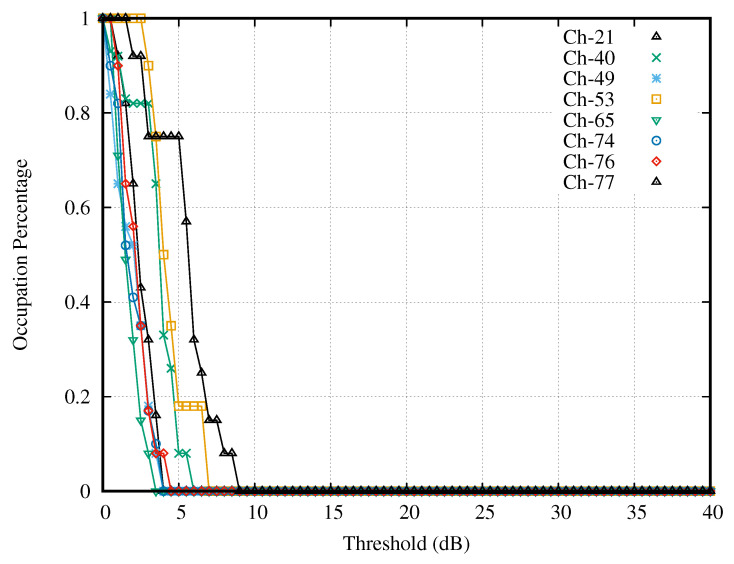
Channels with no activities.

Figure 9 presents the curves of the selected channels with no activity. It is noticeable that all curves drop sharply before the threshold becomes 9 dB. Therefore, the interval of constant occupation percentage was between 9 and 90 dB, which was longer than that of the strong activity case. Accordingly, the search interval of the decision thresholds in the case of channels with no activity was the superset of that of the channels with strong activity, and the search interval of the decision thresholds in the case of channels with strong activity also served as the search interval in the case of channels with no activity.

**Figure 10:**
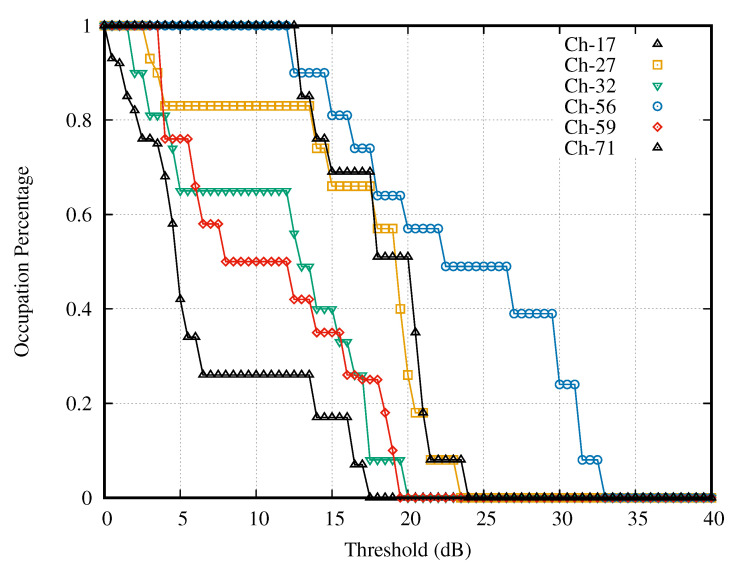
Channels with weak activities.

Figure 10 presents the curves of the selected channels with weak activity. It is noticeable that the intersection of the intervals with constant occupation percentage of all curves is 8–12 dB. Then, the intersection of the interval 8–12 dB and the strong case’s search interval (9–17 dB) was 9–12 dB.

Table 1 presents the number of weak channels that do not have a constant duty cycle at $\gamma_{i}\in \left[9,12\right]$ dB. It is noticeable that the minimum point is at 12.0 dB. Hence, we select 12.0 dB as the appropriate decision threshold and use this threshold for evaluating the spectrum occupancy in the next section.

It can be noticed that only the channels with strong activity have the occupation percentage curves with triple slopes, i.e., two breakpoints. The proposed model analyzes the characteristics of spectrum occupancy by using both breakpoints as the boundary of the particular threshold. Thus, the proposed model will perform well if there are some channels that have strong activity. It should be careful to arrange the measurement setup in order to confirm that the channels having the strong activity are available. For example, the number of the measurement periods should be enough to obtain meaningful measurement data. As another example, the resolution bandwidth should be narrow enough so that the detected signals have high SNR.

### 4.4. Occupancy Classification Criteria

Theoretically, the evaluation of spectrum usage will not have an error if the broadcasting station emits the signal that occupies only the licensed spectrum. But, in practice, the broadcasting station might illegitimately emit the signal that interferes with the licensed spectrum of other broadcasting stations. For example, when the broadcasting station increases the SNR of its channel by emitting a signal with a higher power level, the unwanted emission that interferes with the licensed spectrum of the adjacent channels is not negligible. Then, based on conventional spectrum occupancy evaluation, the interfered channels will be mistakenly considered occupied. To improve the accuracy in evaluating spectrum occupancy, we use the measured power level over the channel’s passband instead of the power level over a whole bandwidth (both passband and guardband) of the channel while determining the occupancy status in Equation (11). In practice, in order to measure the power levels over the channel’s passband and the channel’s guardband separately, it is necessary to employ the spectrum analyzer that can analyze power spectrum over the span (the range between the start and stop frequencies) which is narrower than or equal to the bandwidth of the guardband of a channel. Accordingly, we present how to exploit those measured data for distinguishing the really used channel from the channel occupied by the interference and describe the occupancy classification criteria when evaluating channel utilization.

Firstly, we exploit the measured power level of passband and guardband within the same channel by representing them in two terms: (i) the passband’s duty cycle and (ii) the guardband’s duty cycle. The passband’s duty cycle of Channel *c*, denoted by $D_{c}^{P}$, can be estimated with Equation (10), where the averaged power $P_{av,c,\tau }$ in Equation (12) are determined from the $P_{c,\tau }^{s}$ at the segment $s=2,\cdots ,N_{s}-1$. The guardband’s duty cycle of Channel *c*, denoted by $D_{c}^{G}$, can be estimated with Equation (10), where the averaged power $P_{av,c,\tau }$ in Equation (12) are determined from the $P_{c,\tau }^{s}$ at the segment s=1 and $N_{s}$. Then, we classify all channels, based on the values of $D_{c}^{P}$ and $D_{c}^{G}$, into four classes: (i) used normally, (ii) used abnormally, (iii) unused normally, and (iv) unused abnormally by the following criteria.

- A channel is used normally if $D_{c}^{P}\neq 0$ and $D_{c}^{G}=0$;
- A channel is used abnormally if $0<D_{c}^{G}<D_{c}^{P}$;
- A channel is unused normally if $D_{c}^{P}=0$ and $D_{c}^{G}=0$;
- A channel is unused abnormally if $D_{c}^{P}<D_{c}^{G}$.

### 4.5. Interference Criteria

In practice, the used abnormally and the unused abnormally channels are the channels in which the guardbands are occupied by unwanted emitting signals. Based on the proposed criteria in Section 4.4, the guardbands of the channels that are classified as unused abnormally channels are occupied, but the passbands of those channels are unoccupied. On the other hand, both the guardbands and the passbands of the channels that are classified as used abnormally channels are occupied. In order to mitigate the interferences, the used and unused abnormally channels should be further classified whether they are the sources of interference or not. Since the criteria in the literature do not consider the guardband and the passband separately, we propose the interference criteria to distinguish the channels that are sources of interference from the interfered channels. The criteria consider the power levels within the passband and the guardband.

#### 4.5.1. Interfered Channel Criteria

The channels, which previously classified as unused abnormally, can be further classified into two classes: the channels interfered from adjacent channels, and channels interfered from unknown sources. We observe the differences between the measured power level of Channel *c* and those of two adjacent channels (Channel c−1 and Channel c+1). Firstly, we exploit the measured power levels of passband and guardband within the same channels by representing them in six terms: (i) the passband’s duty cycle of Channel *c* denoted by ($D_{c}^{P}$), (ii) the guardband’s duty cycle of Channel *c* denoted by ($D_{c}^{G}$), (iii) the passband’s duty cycle of Channel c−1 denoted by ($D_{c-1}^{P}$), (iv) the guardband’s duty cycle of Channel *c* denoted by ($D_{c-1}^{G}$), (v) the passband’s duty cycle of Channel c−1 denoted by ($D_{c+1}^{P}$), and (vi) the guardband’s duty cycle of Channel *c* denoted by ($D_{c+1}^{G}$). All six terms are estimated based on Section 4.4 Then, we classify the channels that are unused abnormally, based on the values of $D_{c}^{P}$, $D_{c}^{G}$, $D_{c\pm 1}^{P}$, and $D_{c\pm 1}^{G}$, into two classes: (i) occupied by interference from the adjacent channels and (ii) occupied by unknown signals by the following criteria.

- A channel is interfered from adjacent channels if $D_{c}^{P}\leq D_{c}^{G}\leq D_{c\pm 1}^{G}\leq D_{c\pm 1}^{P}$;
- A channel is interfered from unknown sources if otherwise.

It can be noticed that the criteria can only be used for identifying a channel which is interfered from adjacent channels because the comparison between the duty cycle of the channel and that of adjacent channels must be made. Thus, the criteria do not apply to the case that the channel is interfered from unknown sources.

#### 4.5.2. Interference Source Criteria

The channels, which previously classified as used abnormally, can be further classified into two classes: obvious interference source channels and unobvious interference source channels. We observe the difference between the measured power level of Channel *c* and those of two adjacent channels (Channel c−1 and Channel c+1). Firstly, we exploit the measured power level for passband and guardband within the same channels by representing them in six terms, which are estimated based on Section 4.5.1. Then, we classify the channels that are used abnormally, based on the values of $D_{c}^{P}$, $D_{c}^{G}$, $D_{c\pm 1}^{P}$ and $D_{c\pm 1}^{G}$, into two classes: (i) obvious interference source channels that interfere their adjacent channels and (ii) unobvious interference source channels by the following criteria.

- A channel is an obvious interference source channel that interfere its adjacent channels if $D_{c}^{P}\geq D_{c}^{G}\geq D_{c\pm 1}^{G}\geq D_{c\pm 1}^{P}$;
- A channel is an unobvious interference source channel if otherwise.

It can be noticed that the criteria can only be used for identifying a channel which interferes its adjacent channels because the comparison between the duty cycle of the channel and its adjacent channels must be made. Thus, the criteria do not apply to the case that the channel interferes other channels, which are non-adjacent channels.

## 5. Insight of Spectrum Utilization

This section demonstrates that the evaluation of the spectrum utilization in radio broadcasting can be improved with the proposed measurement strategy and the threshold selection criteria. The proposed measurement strategy yields empirical data, on which the proposed threshold selection criteria are applied to obtain the threshold that gives accurate decisions for the particular situation. In this paper, the particular situation is FM radio broadcasting in the province of Thailand. Moreover, for each channel, decided by the threshold to be occupied, we further classify whether the channel is really used by the broadcasting station of that channel or is simply affected by interferences from other channels. The classification can be done by the proposed criteria. The difficulty of the situation in this paper is that the channels with strong activities are far different from the channels with weak activities in terms of the variance of the measured power. The threshold that gives accurate decisions for the channels with strong activities might not give accurate decisions for the channels with weak activities. The proposed classification criteria can help improve accuracy by evaluating the spectrums of passband and guardband separately. This is also the rationale that our measurement strategy measures the power of passbands and guardbands separately. Based on the proposed measurement strategy in the frequency dimension, we obtained the measured data of 81 channels, consisting of 81 passbands and 81 pairs of guardbands (lower and upper guardbands).

### 5.1. Evaluation of Channel Occupancy

**Figure 11:**
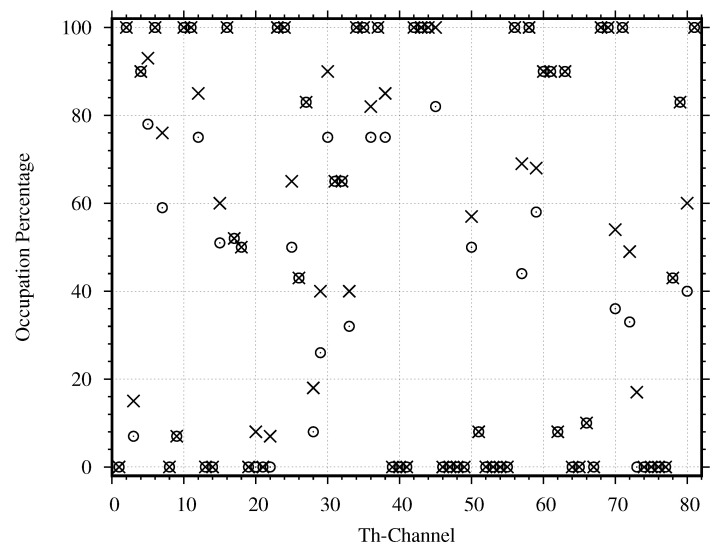
The duty cycle estimated by the proposed threshold (∘: −92.14) is compared with the duty cycle estimated by the ITU threshold (×: −94.14 dBm) for all channels when the power density is considered in a whole channel.

In order to search the occupied channels with the results from the evaluation of channel occupancy, we use the power density in a whole channel to estimate all channels’ duty cycles with the conventional technique. We estimate the duty cycles by using Equations (10)–(12) and plot their values in Figure 11. Based on the evaluation with the ITU threshold, 57 channels have non-zero duty cycles, while 24 channels have zero duty cycle. Based on the evaluation with the proposed threshold, 54 channels have non-zero duty cycles, while 27 channels have zero duty cycle. Therefore, there are 3 channels (Channel 20, 22 and 73) that are considered occupied based on the ITU threshold but are considered unoccupied based on the proposed threshold.

### 5.2. Evaluation of Channel Usage

**Figure 12:**
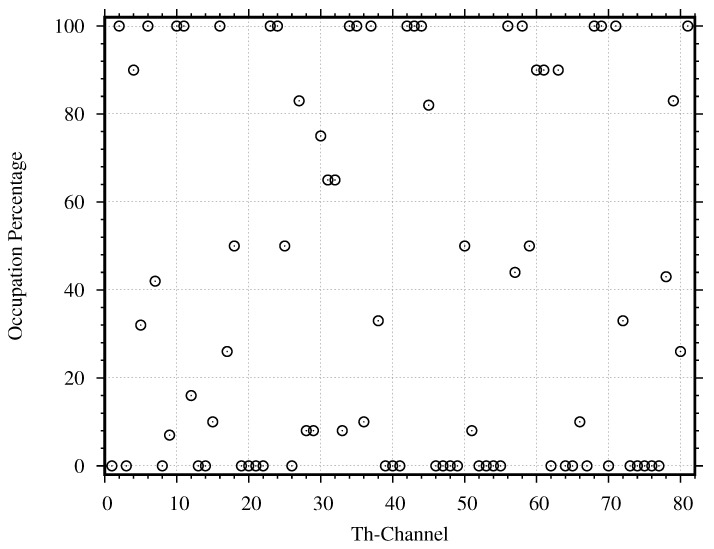
Evaluation of duty cycle with power density in only passband of each channel.

**Figure 13:**
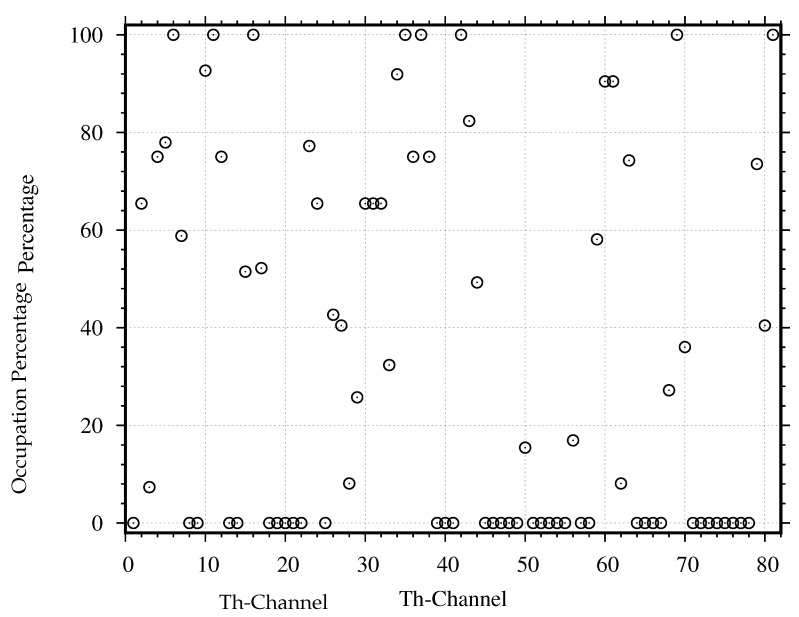
Evaluation of duty cycle with power density in only guardband of each channel.

To further classify the non-zero duty cycles into two groups, namely the used channels and the unused channels, we estimate the duty cycle again, where the passband and guardband of each channel are considered separately. The passband’s duty cycle for each channel $D_{c}^{P}$ can be estimated by substituting the power density in the channel’s passband into Equations (10)–(12) and plot their values in Figure 12. Based on the evaluation with the proposed threshold, 50 channels have non-zero duty cycles, while 31 channels have zero duty cycle. When comparing the whole channel evaluation in Figure 11 with the passband evaluation in Figure 12, we find 7 more unoccupied channels (Channel 3, 20, 22, 26, 62, 70, and 73). Then, we estimate the guardband’s duty cycle for each channel $D_{c}^{G}$ by substituting the power density in the channel’s guardband into Equations (10)–(12) and plot their values in Figure 13. As we expected, the guardband’s duty cycles, which should be zero for all channels, are not zero for many channels. Based on the evaluation with the proposed threshold, 43 channels have non-zero duty cycles, while 38 channels have zero duty cycle. When comparing the whole channel evaluation in Figure 11 with the guardband evaluation in Figure 13, we find 14 more unoccupied channels (Channel 9, 18, 20, 22, 25, 45, 51, 57, 58 66, 71, 72, 73, and 78). Therefore, the proposed threshold, together with the passband and guardband evaluations, can find many more unoccupied channels. Still, not all occupied channels are really used and only get interfered by other channels.

To find all unused channels, we use the proposed criteria to classify the used and unused channels as described in Section 4.4 The criteria compare the duty cycle $D_{c}^{P}$ in Figure 12 with $D_{c}^{G}$ in Figure 13. Based on the obtained $D_{c}^{P}$ and $D_{c}^{G}$, we found 11 channels that are used normally, 28 channels that are used abnormally, 27 channels that are unused normally, and 15 channels that are unused abnormally. Therefore, the proposed criteria can find 42 unused channels. Compared to the conventional method that finds only the unoccupied channels, using the proposed criteria to find the unused channels can find 18 more channels.

### 5.3. Interfered Channels

To further identify the channels that are unused but are occupied by interference, we use the proposed criteria in Section 4.5.1 and the duty cycle estimated in Section 5.2. Based on the obtained ($D_{c}^{P}$ and $D_{c}^{G}$), we found 15 channels (Channel 3, 5, 7, 12, 15, 17, 26, 29, 33, 36, 38, 59, 62, 70, and 80) that are interfered by their adjacent channels. Hence, these channels are actually unused, and will be available for allocation if the interferences are mitigated.

### 5.4. Interference Source Criteria

To further identify the channels that interfere with adjacent channels and cause an error when evaluating the spectrum occupancy, we use the proposed criteria in Section 4.5.2 and the duty cycle estimated in Section 5.2. Based on the obtained ($D_{c}^{P}$ and $D_{c}^{G}$), we found 16 channels (Channel 2, 4, 6, 11, 16, 30, 32, 34, 35, 37, 60, 61, 63, 69, 79 and 81) that interfere their adjacent channels in both guardbands and passbands. They are previously classified as the abnormally used channels, while other 12 abnormally used channels are inobvious interference source channels because the observable power levels in the guardband of their adjacent channels are not certainly from them. Hence, if those 16 interference sources can mitigate their emitting interference, their adjacent channels will be available for allocation.

## 6. Conclusions

This paper presents the spectrum occupancy model for suburban environments to investigate the unused channels in the spectrum of FM radio broadcasting. The model is composed of three components: (i) the spectrum measurement method, (ii) the appropriate decision threshold, and (iii) the criteria for channel classification. To validate the proposed model, the obtained results were compared with that obtained from the conventional model. Both conventional and proposed models were experimented in the FM radio broadcasting network in a province of Thailand to classify the unused channels from all 81 licensed channels. The selected area has suburban environments, and interferences are frequently found according to our preliminary measurement results. Conventionally, the channels are simply classified as occupied or unoccupied channels. The opportunities for spectrum sharing are quite limited because some channels, classified as occupied, are actually unused. This can happen when the unused channels get interfered from adjacent analog broadcasting channels. On the other hand, the proposed criteria for channel classification can distinguish the channels that are really used from the channels that are actually unused. For example, in the demonstrated case, the conventional classification technique found 24 unoccupied channels, while the proposed classification technique found 42 unused channels. Since the proposed model can distinguish the unused channels from the channels that are occupied only by interference, they can be adopted in sensing systems of the networks with spectrum sharing, for example, digital audio broadcasting (DAB) and dynamic spectrum access (DSA).

Furthermore, this paper presents interference criteria to classify the abnormally used/unused channels into the channels, interfered from adjacent channels, and the channels that are obviously the sources of interference. The criteria found 15 interfered channels and 16 channels that are the sources of interference. These 15 channels are unused but have interference. Thus, before assigning them for secondary users, we should prepare a plan to mitigate the interference. In addition, the knowledge of those 16 channels, which are the sources of interference, are useful information for cognitive radio networks to avoid assigning their adjacent channels to secondary users.

## Figures and Tables

**Table 1:** The number of channels whose duty cycles are not constant at $\gamma_{i}$.

$\gamma_{i}$	$K_{c}\left(\gamma_{i}\right)$
9.0	28
9.5	26
10.0	29
10.5	27
11.0	25
11.5	24
12.0	20
12.5	27

## Data Availability

Data is contained within this article.
